# Temperature-Responsive Ionic Liquids: Polymer Structure, Phase Transition Behaviors and Applications

**DOI:** 10.3390/polym18192392

**Published:** 2026-09-30

**Authors:** Lijun Guo, Xiaoying Ji, Jian Zhou, Yanju Liu, Wenhua Zhang, Xin Huang, Dongliang Li, Bi Shi

**Affiliations:** 1 China Tobacco Sichuan Industrial Company, Ltd., Chengdu 610065, China; 2 Key Laboratory of Leather Chemistry and Engineering of Ministry of Education, Sichuan University, Chengdu 610065, China

**Keywords:** temperature-responsive, ionic liquids, polymer structure, fundamental behaviors, applications

## Abstract

Temperature-responsive ionic liquids (TRILs) represent a significant advance of smart functional materials with thermally tailorable phase transition behaviors. TRILs exhibit typical lower critical solution temperature (LCST) and upper critical solution temperature (UCST) liquid–liquid phase transitions, as well as versatile liquid–solid transition modes involving crystallization, precipitation, and liquid crystal formation, which endow TRILs with superior tunability inaccessible to conventional solvents and ordinary ILs, enabling reversible regulation of solubility, miscibility, ion transport, and interfacial properties under temperature stimuli. This review systematically elaborates the structural characteristics of TRIL cations and anions, clarifies the intrinsic mechanisms of thermally triggered phase transitions, and comprehensively summarizes state-of-the-art advances in multidisciplinary applications covering water treatment, gas separation, controlled drug delivery, switchable catalysis, and electrochemical energy storage. We fully discuss the structural advantages and working principles of TRIL-based smart systems, and analyze the current bottlenecks including high intrinsic viscosity, limited regulation range, ambiguous biocompatibility, and inadequate long-term cycling stability. Future research directions are further proposed to guide the rational molecular design, composite modification, and multi-stimuli integration of high-performance TRIL materials. This work aims to provide a comprehensive and systematic reference for the development and industrial translation of next-generation thermoresponsive functional materials with case-specific environmental advantages.

## 1. Introduction

Due to the intriguing physicochemical properties, such as ultralow vapor pressure, non-flammability, excellent thermal stability, and wide electrochemical windows, ionic liquids (ILs) are often considered as alternative media with low volatility and tunable properties, but their environmental safety and sustainability should be assessed case by case rather than assumed from low volatility alone [1,2], and they possess outstanding structural and functional tunability, holding great promise for applications in separation engineering, catalysis, biomedicine, and energy devices [3,4]. However, pristine ILs lack active environmental responsiveness, which restricts their intelligent modulation capability and hinders their further development in adaptive, switchable functional platforms [5].

Compared with thermoresponsive polymers, such as PNIPAM, PVCL, and POEGMA, TRILs offer additional advantages such as negligible volatility, high ionic conductivity, wide electrochemical windows, and tunable liquid–liquid/liquid–solid transitions [6,7]. By rationally tailoring cationic alkyl chains, anion species, and functional substituents, the delicate balance of hydrogen bonding, electrostatic forces, van der Waals interactions, and hydrophobic effects can be dynamically regulated [8,9,10], thus enabling liquid–liquid/liquid–solid transitions and realizing on-demand functional switching of smart materials. For example, their reversible phase separation enables switchable extraction and cyclic regeneration in water treatment and gas separation, which effectively reduces energy consumption and improves separation efficiency [11,12,13]. In biomedical fields, TRILs overcome the inherent limitations of conventional thermosresponsive hydrogels, achieving efficient solubilization of poorly soluble drugs and precisely controllable drug release with enhanced biostability [14,15]. In catalytic systems, TRIL-based thermomorphic biphasic platforms integrate the merits of homogeneous and heterogeneous catalysis, realizing high-efficiency catalytic reactions and facile catalyst recycling [16,17]. More notably, emerging applications in electrochemical energy storage enable thermal adaptive self-protection and visual state monitoring, which effectively mitigate the thermal runaway risks of advanced energy storage devices [18,19,20].

Existing reviews have primarily focused on applications of ILs in individual fields, while few systematic summaries concentrate on the intrinsic phase transition mechanisms and multi-scenario applicability of TRILs [21,22]. Herein, this review comprehensively elucidates the structural composition characteristics of TRILs, systematically classifies their liquid–liquid and liquid–solid phase transition mechanisms, and summarizes the latest research progress in water purification, gas separation, controlled drug delivery, switchable catalysis, and smart energy storage [23,24]. Current technical bottlenecks and future development trends are also thoroughly discussed, aiming to provide a solid theoretical basis and practical guidance for the design, modification, and industrial promotion of high-performance thermosresponsive ionic liquid materials.

## 2. Molecular Structure of TRILs

**Figure 1:**
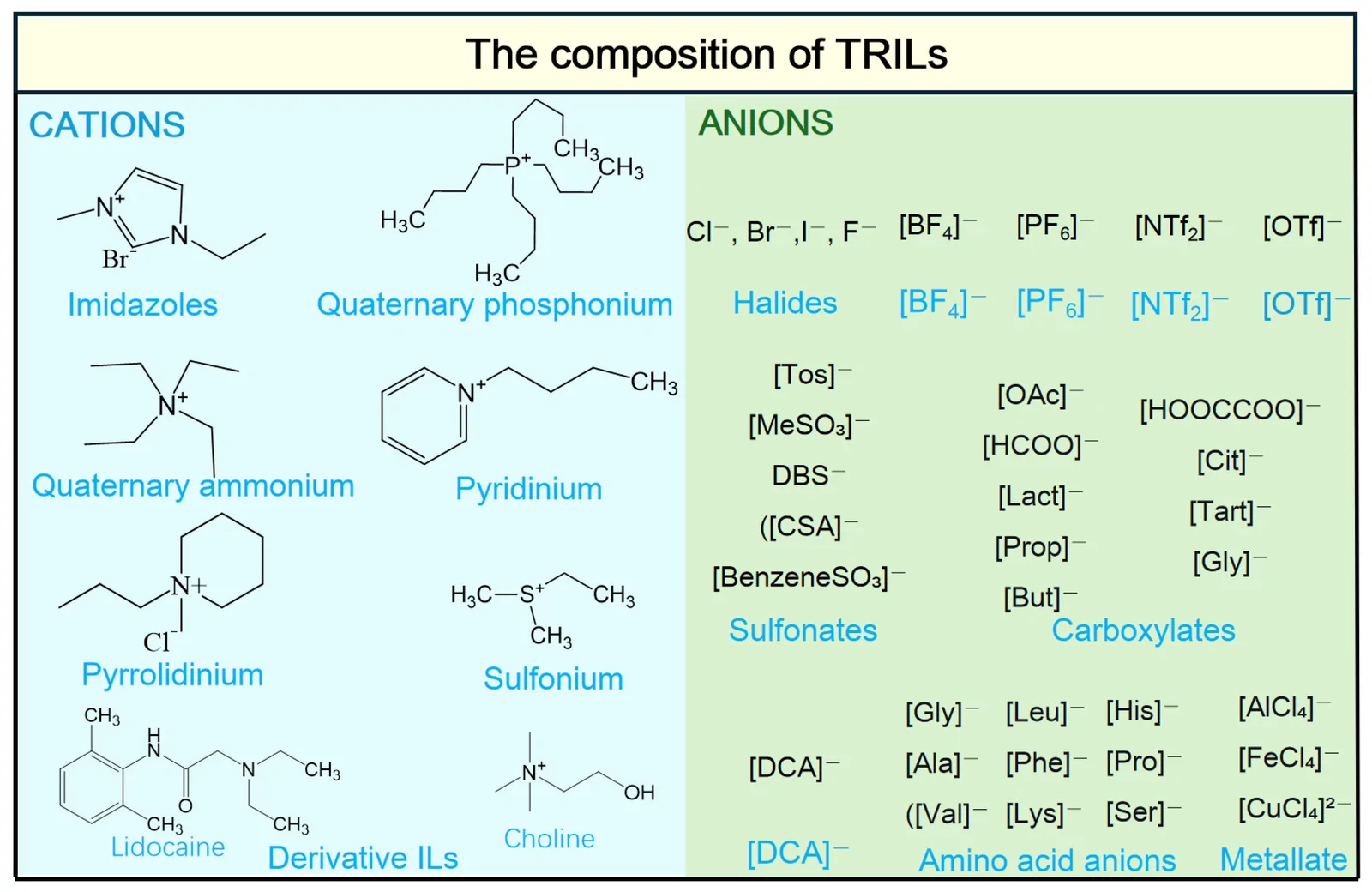
Composition of TRILs.

TRILs are defined as a subclass of ionic liquids (ILs) that exhibit reversible temperature-responsive phase transitions, including LCST/UCST liquid–liquid transitions and liquid–solid transitions, such as precipitation, crystallization/melting, ionic liquid crystal (ILC) formation, and ionic plastic crystal (IPC) formation. TRILs are composed of ions and are therefore small-molecule ILs or ionic liquid mixtures, and they are not polymers, hydrogels, or ionogels [25,26]. The most widely adopted TRILs consist of cations paired with diverse organic and inorganic anions. The chemical structures and corresponding categories of frequently used cations and anions in TRILs are presented in Figure 1. The diverse properties of TRILs, especially their temperature-responsive nature, originate from the identity and interaction of their constituent ions, including electrostatic attraction, hydrogen bonding, van der Waals forces, and hydrophobic/hydrophilic interactions [27,28,29].

### 2.1. Cations

Cations constitute the structural backbone of TRILs and exert a dominant influence on their physicochemical properties and temperature-responsive behavior [30,31]. The most commonly employed cations, such as imidazolium, pyridinium, quaternary ammonium, and quaternary phosphonium species, are typically characterized by bulky, asymmetric molecular architectures and weak coordination tendencies [32]. Cations can effectively regulate the phase state of TRILs because structural asymmetry can strongly frustrate crystal packing and significantly lower the melting point, enabling these salts to undergo reversible transitions among solid, liquid, and gaseous states at specific temperatures [33,34]. In addition, the cation architecture provides a versatile platform for property modulation. Altering the alkyl substituents allows for precise control over hydrophobic/hydrophilic balance, polarity, viscosity, and the temperature at which phase transitions occur, whereas the incorporation of functional groups (e.g., -OH, -COOH, -NH_2_) not only imparts specific chemical reactivity but also confers stimuli-responsive features, including thermoresponsiveness, making the resulting TRILs amenable to diverse applications [35,36]. Consequently, the judicious selection of cations allows for the rational design of TRILs with tailored temperature-responsive properties, which has driven their extensive application across diverse fields. For instance, NASA TechPort (2019) designed a cation-modified IL heat exchanger that remains solid and converts into a liquid upon heat absorption, further promoting the development of heat-resistant materials for extreme environments [37].

### 2.2. Anions

Anions are equally critical in shaping the physicochemical properties and temperature-responsive behavior of TRILs, complementing the role of cations. The most widely investigated anions include halides (e.g., Cl^−^, Br^−^), tetrafluoroborate ([BF_4_]^−^), hexafluorophosphate ([PF_6_]^−^), bis(trifluoromethanesulfonyl)imide ([NTf_2_]^−^), trifluoromethanesulfonate ([OTf]^−^), carboxylates (e.g., [OAc]^−^), and amino acid-derived anions, as shown in Figure 1 [38,39,40,41]. The structural attributes of anions, such as ionic size, charge distribution, and coordination capacity, directly govern key performance indicators of TRILs, including melting point, viscosity, hydrophilicity, thermal stability, and phase-transition temperature. Anions with small size and strong coordinating capacity (such as halides) increase viscosity and hydrophilicity, while the hydrophilic or hydrophobic nature of anions determines their miscibility with water and other solvents and thus their temperature-dependent phase behavior. In addition, anions such as [NTf_2_]^−^ and [OTf]^−^ offer wide electrochemical windows up to 6 V and exhibit high electrochemical and thermal stability [42,43,44]. Based on these characteristics, anion selection enables tailored applications of TRILs. [PF_6_]-based TRILs are preferred for liquid–liquid extractions due to their hydrophobicity; carboxylate and halide systems excel in dissolving cellulose and lignocellulosic biomass; [NTf_2_]^−^-based TRILs are indispensable in lithium batteries and supercapacitors because of their high stability and low viscosity; and amino acid anions, being biocompatible and chiral, find use in drug delivery and enantioselective separation [45,46,47].

## 3. Phase Transition Behaviors of TRILs

The defining characteristic of TRILs is their ability to undergo a reversible macroscopic phase transition in response to a change in temperature, which is a manifestation of the delicate balance between various highly temperature-dependent intermolecular interactions, including hydrogen bonding, electrostatic forces, van der Waals interactions, and hydrophobic effects [45,48]. The phase transition of TRILs is triggered when a specific temperature threshold, known as the critical solution temperature, is reached, and can be categorized into liquid–liquid transitions and liquid–solid transitions, depending on the nature of the phase separation and the resulting products [13,46,49].

### 3.1. Liquid–Liquid Transition

**Figure 2:**
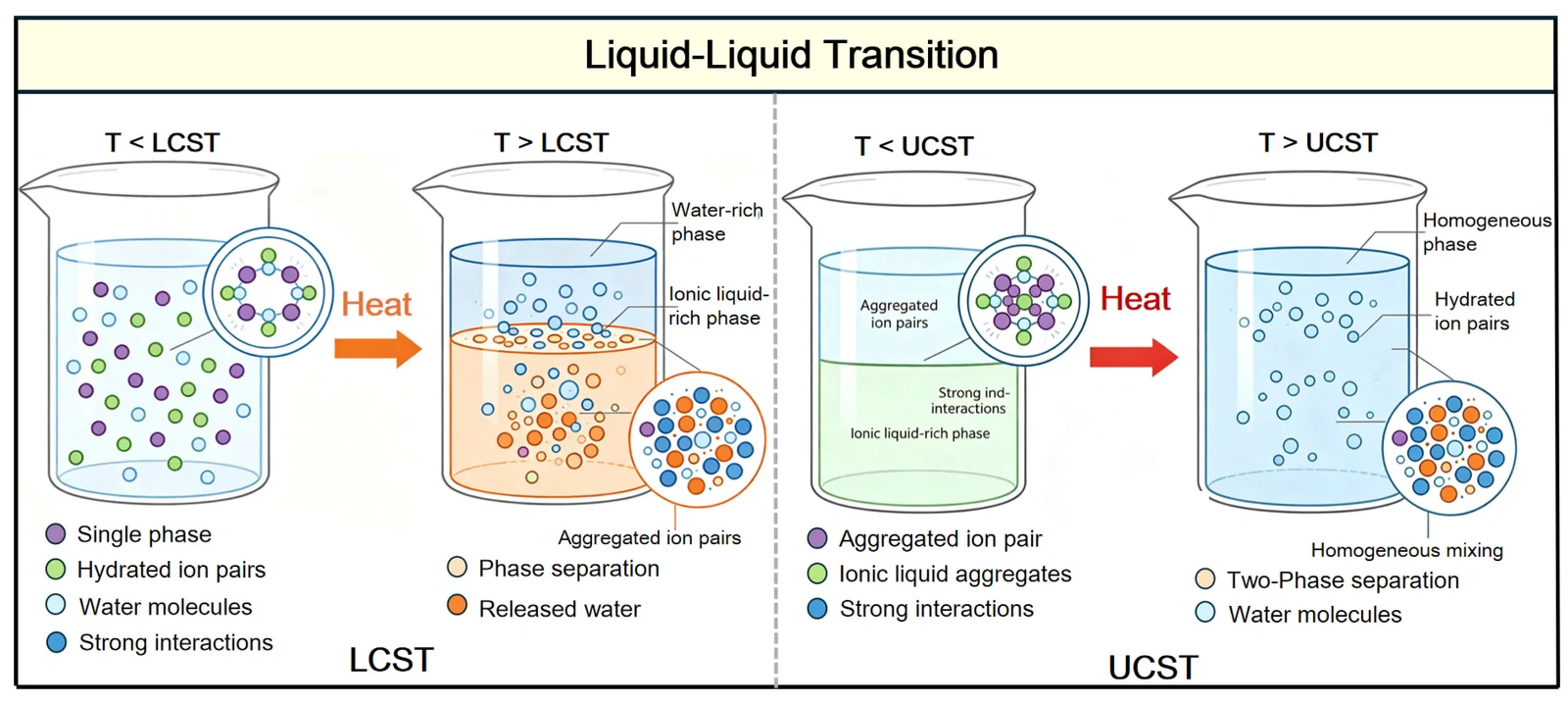
The process of liquid–liquid transition.

The liquid–liquid phase transition is the most extensively studied phenomenon in TRILs, which is characterized by a homogeneous solution separating into two distinct, immiscible liquid phases upon a change in temperature [47,50,51,52]. This behavior is typically observed in aqueous solutions of TRILs and is fundamentally governed by the temperature-dependent solubility of the ILs in water. Based on the direction of the phase change with respect to temperature, lower critical solution temperature (LCST) and upper critical solution temperature (UCST) behavior for the liquid–liquid phase transition have been found, as shown in Figure 2 [23,32,53,54].

LCST behavior is the most common and technologically relevant type of liquid–liquid transition for TRILs. At a specific temperature, known as the LCST, the ionic liquid and water are completely miscible, forming a single homogeneous phase. Upon heating above the LCST, the ordered hydration shell is disrupted, releasing water molecules into the bulk and leading to a net increase in entropy, which favors phase separation and convents into a water-rich phase and an ionic-liquid-rich phase [50,51,55].

UCST behavior is the thermodynamic opposite of LCST, which is highly rare. When the temperature is lower than the UCST, the strong attractive interactions (e.g., hydrogen bonds or electrostatic attractions) between the ionic liquid and water or between ionic liquid ions themselves are favored, leading to the formation of aggregates and phase separation. Upon heating above the UCST, thermal energy overcomes these attractive forces, allowing for complete miscibility [56,57].

Notably, the liquid–liquid phase transition of ILs exhibits a high degree of tunability and designability [58]. For instance, tributylmethylphosphonium methylsulfate ([P_4441_][MeSO_4_]) is well-known for its LCST-type behavior in water, and its phase transition temperature can be finely tuned by altering the alkyl chain length on the cation or by changing the anion [59]. The ability to precisely control the phase separation temperature is crucial for applications such as extraction, catalysis, and water treatment, where a reversible switch between a single-phase and a biphasic system is highly required.

The LCST/UCST behavior of TRILs depends not only on ionic structure but also on external variables. Concentration is a key factor because UCST systems are strongly concentration-dependent and often exhibit a critical composition, while LCST cloud points can also shift with concentration in TRIL-water mixtures. Water content is equally important. In aqueous TRILs, water hydrates ions, forms hydrogen-bond networks, and changes the effective TRIL concentration; increasing water content can therefore shift LCST/UCST. In non-aqueous or ionogel systems, residual water acts as an impurity or plasticizer that alters ion pairing, transition temperature, conductivity, and stability. Salts and ionic strength introduce Hofmeister effects and electrostatic screening. Salting-out ions such as SO_4_^2−^, HPO_4_^2−^, and F^−^ enhance hydrophobic effects and typically lower LCST, whereas salting-in ions such as SCN^−^, I^−^, and ClO_4_^−^ increase solubility and raise LCST. For UCST systems driven by attractive electrostatic interactions, increasing ionic strength screens these interactions and can lower or eliminate UCST. Therefore, LCST/UCST values should be reported together with concentration, water content, salt type/concentration, and ionic strength.

### 3.2. Liquid–Solid Transition

**Figure 3:**
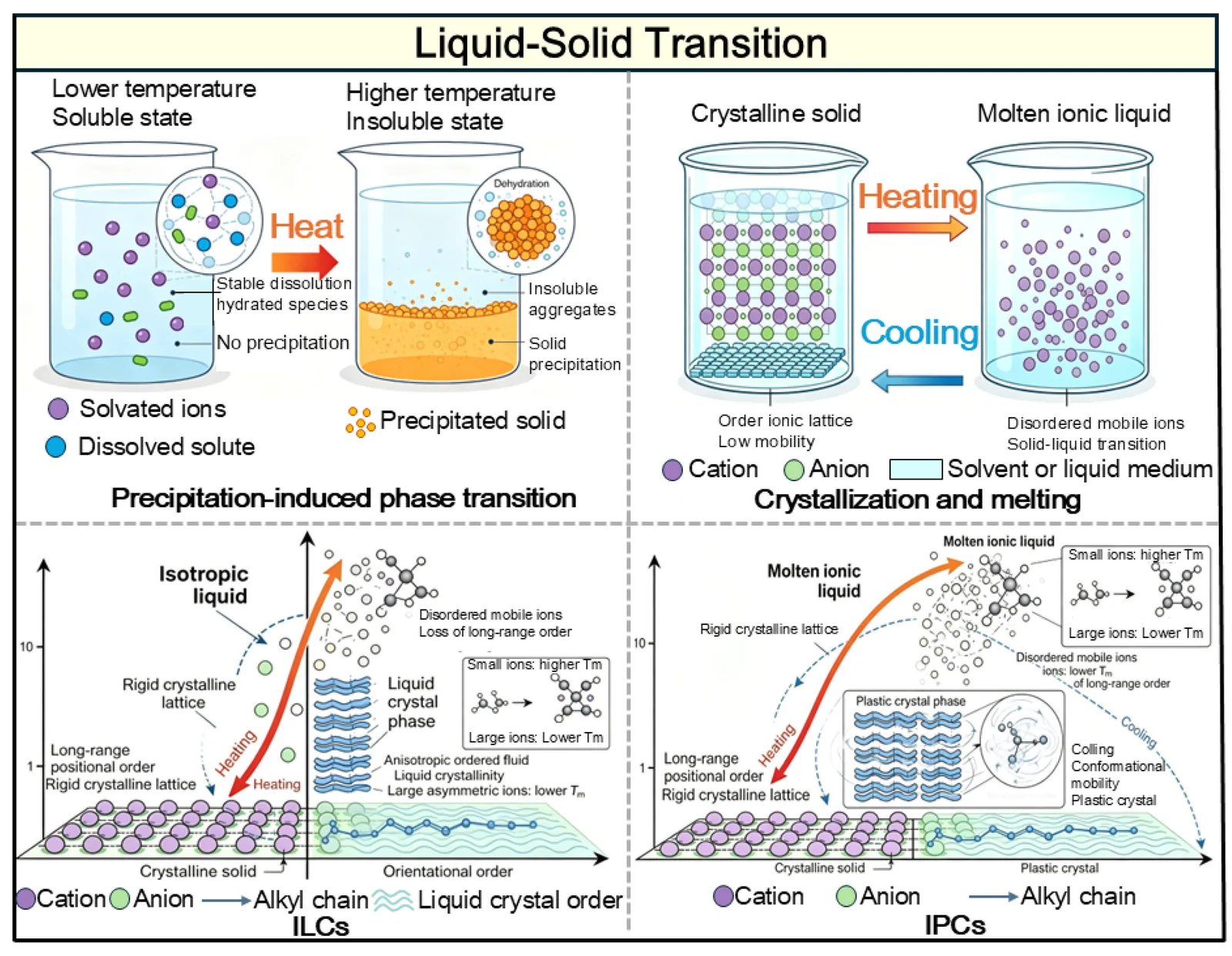
The process of liquid–solid transition.

The liquid–solid phase transition involves a change in the physical state of one or more components in the system, where a liquid phase transforms into a solid phase upon a change in temperature [11,12,40,47]. Based on the principle of solid phase formation, liquid–solid phase transitions can be categorized into precipitation-induced phase transitions, crystallization and melting, formation of ionic liquid crystals (ILCs), and ionic plastic crystal phases (IPCs), as shown in Figure 3.

For the precipitation-induced phase transition, the TRIL or solute becomes insoluble and precipitates out of the solution as a solid upon a temperature change [47,52,60]. For example, Wang et al. reported an amine-functionalized IL, which can chemically absorb CO_2_ to form a solid carbamate salt that can be easily separated from the liquid phase by filtration. When the temperature increases to 60 °C, the solid carbamate decomposes can release the CO_2_ and regenerate the IL. This phase transition process is reversible. Hence, the precipitation-induced phase transition of ILs is widely used for gas capturing and separation [61].

Crystallization and melting are the most fundamental liquid–solid transitions, referring to the melting (solid to liquid) and crystallization (liquid to solid) of TRILs, and are driven by temperature variation [62,63,64]. At low temperature, cations and anions assemble into ordered crystals and exhibit a solid state through electrostatic interactions and hydrogen bonds. When the temperature rises above the melting point, thermal motion disrupts the crystal lattice, and ions transform into a freely mobile liquid state. During the cooling process, most TRILs are susceptible to supercooling and tend to form glassy states rather than crystals, due to the asymmetric ion size, high degree of charge delocalization, and high flexibility of the alkyl chains [10,65,66]. Therefore, the crystallization and melting behavior of TRILs can not only be regulated by the type and size of the anions and cations, but can also be designed by altering ionic symmetry, alkyl chain length, the strength of intermolecular interactions, and the rate of heating and cooling.

TRILs with long alkyl chains can form ILCs under temperature variation. ILCs are a subclass of ionic soft materials that combine the properties of ILs with liquid crystalline order. They exhibit one or more liquid crystalline mesophases, either thermotropic or lyotropic, and therefore possess both ionic conductivity and anisotropic ordering. In detail, cations and anions construct compact regular crystals via electrostatic interaction and hydrophobic stacking of alkyl chains, and show a crystalline solid state at low temperature [67,68,69]. When heated to the crystal-mesophase transition temperature, long-range positional order of crystal lattices collapses, while long alkyl chains of ions retain oriented arrangement, thus turning the system into a liquid crystal phase, which combines the anisotropy of crystalline solids and the fluidity of liquids. Further heating up to a clearing point, the orientational order of alkyl chains is completely eliminated, yielding a conventional isotropic liquid. More importantly, the liquid-crystalline phase of TRILs can be regulated by molecular structure (e.g., increasing alkyl chain length, adopting asymmetric ions, enhancing charge delocalization, and introducing stacking-prone functional groups), temperature, and solvent species [70]. Hence, the unique ordered nanostructure and excellent tunability of ILCs make them promising materials for applications in sensors, electrolytes, and templates for nanomaterial synthesis.

IPCs, sometimes called organic ionic plastic crystals (OIPCs), are ionic solids that exhibit a plastic crystalline phase driven by temperature variation [71]. At low temperatures, cations and anions pack into rigid and well-ordered crystals. Upon heating to the solid–solid transition temperature, the mass centers of ions retain long-range positional order within the lattice, while cations and anions undergo rapid three-dimensional rotation and orientational disorder, generating the plastic crystal phase. Further heating to the melting point breaks the lattice framework, yielding isotropic molten ionic liquid [72]. Hence, IPCs formation is always found in ILs with near-spherical and relatively symmetric geometry. Due to the solid-state form, ionic mobility, and plasticity, IPCs are primarily targeted at applications in solid-state electrochemical energy storage, solid-state refrigeration, stimulus-responsive sensing, selective separation, and microelectronic devices [73].

In conclusion, ILs are salts composed entirely of ions with melting points typically below 100 °C. ILCs are ionic mesogens that exhibit liquid crystalline mesophases, combining ionic conductivity with anisotropic order. IPCs are ionic solids with long-range positional order but significant orientational/rotational disorder, and they are mechanically plastic and can exhibit solid-state ionic conductivity. Ionic polymers are macromolecules bearing covalently bound ionic groups, including ionomers, polyelectrolytes, and polymerized ionic liquids (PILs). Hydrogels are three-dimensional polymer networks swollen with water. They are not ILs; however, they become related to ILs when ILs are incorporated as additives/electrolytes or when the polymer network itself contains ionic liquid moieties. If the liquid phase is an IL rather than water, the material is commonly termed an ionogel. In the present system, water plays multiple roles: it acts as a swelling agent and plasticizer, participates in hydrogen bonding and ion solvation, and provides a medium for ion transport. Its content can therefore strongly affect ion pairing, conductivity, mechanical properties, and phase stability. In IL/ILC/IPC systems, water is often present as a controlled component or impurity and can alter mesophase/phase behavior and transport.

### 3.3. Relationship Between Ionic Structure and Phase-Transition Temperature

The phase-transition temperature of TRILs is governed by the balance among electrostatic interactions, hydrogen bonding, van der Waals forces, and hydrophobic effects. Cation structure plays a dominant role. Longer alkyl chains increase hydrophobicity and generally lower the LCST in LCST-type TRILs, while bulky asymmetric cations frustrate crystal packing and shift liquid–solid transitions. Cation headgroups such as imidazolium, pyridinium, quaternary ammonium, and phosphonium differ in charge delocalization, aromaticity, and hydrogen-bonding ability, thereby tuning transition temperatures. Functional groups such as -OH, -COOH, and -NH_2_ strengthen ion-water hydrogen bonding and tend to raise LCST, and they can also introduce UCST behavior. Anion structure is equally important. Hydrophilic anions such as Cl^−^ and [OAc]^−^ strengthen ion-water interactions and tend to increase LCST, whereas hydrophobic anions such as [NTf_2_]^−^ and [PF_6_]^−^ weaken water miscibility and tend to decrease LCST or induce phase separation [70]. For UCST-type systems, the effect is more system-specific: structural changes that strengthen ion–ion or hydrogen-bonding interactions tend to raise UCST, while those that strengthen ion–water interactions tend to lower it [74].

## 4. Applications of Temperature-Responsive Ionic Liquids

Representative TRIL-based applications are summarized in Table 1 [13,68,75,76,77,78,79,80,81,82,83,84,85,86]. TRILs offer intrinsic advantages over conventional thermoresponsive polymers such as PNIPAM, PVCL, and POEGMA. First, in electrochemical energy storage, TRILs can simultaneously serve as electrolytes and thermal switches because of their high ionic conductivity and wide electrochemical windows, whereas conventional thermoresponsive polymers usually require aqueous media and exhibit low ionic conductivity. Second, in gas separation and catalysis, TRILs can act as solvents, absorbents, and catalysts, enabling temperature-switchable homogeneous reactions and phase-separation recovery; thermoresponsive polymers generally serve only as supports or triggers. Third, in water-free or low-water environments, TRILs avoid the freezing, evaporation, and dehydration problems of aqueous polymer systems. Fourth, ILC- and IPC-forming TRILs provide solid-state or anisotropic ionic conduction, which is difficult to achieve with conventional thermoresponsive polymers. Fifth, the phase-transition temperature of TRILs is primarily governed by cation/anion structure rather than molecular weight, enabling more predictable and reproducible transitions. However, real advantages must be demonstrated case by case. Remaining bottlenecks include high viscosity, potential toxicity, poor biodegradability, leakage, broad phase-transition ranges, and limited long-term stability.

### 4.1. Water Treatment

TRILs, as a novel solvent with temperature-triggered phase-change properties, have effectively overcome the bottlenecks of traditional processes thanks to their low volatility, structural design flexibility, reversible phase transitions, and high extraction efficiency, making them a hot topic of research in the field of water treatment. Initially, TRILs are completely miscible with water, forming a homogeneous system that allows for thorough contact with waterborne pollutants at room temperature. Then, phase separation rapidly occurs, and it spontaneously stratifies into a water-rich phase and an ionic liquid-rich phase to realize the separation of solvent and water when the temperature deviates from the critical value [75]. In addition, TRILs can be regenerated using only low-temperature waste heat, resulting in significantly reduced energy consumption and superior cycle stability [85].

TRILs are primarily focused on forward osmosis desalination and concentration of high-salinity wastewater [76]. Traditional forward osmosis technology suffers from high regeneration costs for the draw solution and low recovery rates [77]. In contrast, TRILs can serve as a highly efficient, recyclable draw solution, providing a high osmotic driving force at room temperature to increase water flux relies on the natural osmotic pressure difference across the membrane as the driving force, eliminating the need for external pressure. When heated above the critical temperature, phase separation occurs, enabling the regeneration and reuse of the draw solution. Durling et al. reported a thermosresponsive ionic liquid and observed that [choline][NTf_2_] and the [choline]-derived multicomponent IL mixtures comprising 5:1 and 3:1 ratios of [NTf_2_]/[OH] exhibited significantly higher freshwater yields and enhanced overall desalination efficiency (~0.3%/°C), compared to the other IL mixture, thereby significantly proving the high-efficiency capacity of TRILs for directional desalination [13]. For high-salinity wastewater concentration, TRILs can efficiently bind to free water molecules in high-salinity wastewater, continuously removing water from the system, finally separating water and salt and enabling high-ratio concentration of the wastewater at low temperature. Upon heating to the critical temperature, the system separates into layers, allowing the concentrated high-salinity mother liquor to be directly separated and collected. After desorption, TRILs can be directly regenerated and reused, thereby avoiding the high energy consumption associated with traditional evaporation concentration and the scaling issues common in high-salinity reverse osmosis operations [79].

In addition, TRILs have demonstrated outstanding performance in the removal of toxic metals and organic pollutants, which can avoid the issues of volatile pollution from traditional organic solvents and chemical waste. Efficient pollutant enrichment can be achieved by TRILs through homogeneous extraction, and following realizing pollutant recovery and ionic liquid regeneration via thermal phase transition. Furthermore, TRILs coupled with a hydrogel-modified composite thermosresponsive system can effectively stabilize water flux, enhance water purification precision, and are well-suited for treating wastewater containing complex components. Sun et al. adopted [Bm]Br_2_ to synthesize PAM/AA/[Vim]Br_2_ and used it as an adsorbent for various heavy metal ions, such as Cr, Cu, Zn, and Ni, from water [76]. Hence, TRILs holds broad application prospects in the field of water purification.

### 4.2. Gas Separations

Gas separation plays an important role in chemical engineering, energy and environmental protection, and carbon resource utilization. Functionalized ILs, as a novel functional medium composed of organic cations and inorganic/organic anions that exist in a liquid state at room temperature, offer outstanding advantages for gas separation, such as near-zero volatility, excellent thermal stability, high structural design flexibility, high gas dissolution selectivity, and recyclability [80]. Most importantly, temperature plays a decisive role in TRIL-based gas separation. First, temperature strongly affects gas solubility and diffusivity in TRILs. For physical absorption, gas solubility generally decreases with increasing temperature, while diffusivity and mass transfer are enhanced. Secondly, temperature alters IL viscosity and ion pairing, thereby changing gas transport and interfacial mass transfer. Thirdly, for chemically absorbing TRILs, temperature governs the equilibrium between gas TRIL abduct formation and decomposition, moderating temperatures favor capture, whereas elevated temperatures promote desorption and TRIL regeneration, which is essential for low-energy regeneration. Fourthly, temperature can trigger LCST/UCST or dissolution–precipitation transitions in TRILs, enabling switchable homogeneous capture and phase-separation recovery. Fifthly, temperature affects the selectivity of TRILs. For CO_2_/CH_4_, CO_2_/N_2_, SO_2_/CO_2_, and olefin/paraffin separations, the relative solubility and π-complexation interactions are temperature-dependent. Therefore, optimizing the operating temperature is critical to balance capacity, selectivity, mass transfer, and regeneration energy. In this section, representative studies are discussed with explicit attention to their temperature windows and temperature-dependent separation performance.

The separation of acidic gases by TRILs is currently the most extensively studied. Flue gas from coal-fired power plants, natural gas, and coal chemical process off-gases commonly contain acidic impurity gases, such as CO_2,_ SO_2_, and H_2_S, which not only result in equipment corrosion and reduced calorific value, but also contribute to the greenhouse effect and air pollution, creating an urgent need for highly efficient removal and resource recovery technologies [81]. Traditional alcohol amine absorbents suffer from inherent drawbacks such as high volatility, degradation and failure, severe equipment corrosion, and high energy consumption during regeneration. In contrast, amino and carboxyl, and ether-functionalized TRILs can significantly enhance the adsorption capacity and separation selectivity of acidic gases via a reversible chemical adsorption process, while exhibiting virtually no adsorption toward weakly adsorbable inert gases such as N_2_ and CH_4_, thereby enabling highly efficient and precise separation [82]. Jiang et al. reported that amino-functionalized ILs exhibit excellent selective CO_2_ capture capacity by introducing [Bmim][BF_4_] as a promoter, and the results that the addition of [Bmim][BF_4_] not only realized efficient CO_2_ capture at high temperature (up to 90% CO_2_-rich phase percentage), but also significantly reduced the regeneration energy consumption at low temperature [77]. In addition, ILs containing sulfonic acid groups and ether bonds exhibit unique selective recognition capabilities for SO_2_ and H_2_S, enabling the thorough removal of low-concentration sulfides from industrial exhaust gases and making them suitable for scenarios requiring precise purification of industrial exhaust gases.

ILs also offer significant advantages in the separation of organic gas mixtures and noble gases due to their unique solubility selectivity [84]. The separation of organic gas mixtures via traditional distillation processes, such as low-carbon alkanes and alkenes, which exhibit similar physical properties, always consumes an extremely high amount of energy. ILs enable efficient separation of low-carbon alkanes and alkenes under mild conditions and significantly reduce energy consumption, due to the high solubility of ILs for alkenes via the effects of π-π stacking, electrostatic interactions, and hydrogen bonding, which is far higher than that for saturated alkanes [85]. Although ILs have demonstrated significant technical advantages in the field of gas separation, there are still many bottlenecks hindering their large-scale industrial application at this stage, such as highly complex environments, expensive raw materials, and inferior dynamic adsorption rates, making it difficult to meet the demands of large-scale industrial loading and use [86].

### 4.3. Drug Delivery

It is important to distinguish TRIL-based drug delivery from ordinary IL-based delivery. Ordinary IL-based delivery systems use ILs as solvents, solubilizers, penetration enhancers, microemulsion phases, or stabilizing agents, but do not rely on a temperature-induced phase transition for drug release. In contrast, TRIL-based delivery systems exploit LCST/UCST or liquid–solid transitions to trigger reversible drug encapsulation, release, or carrier separation [87]. Thermoresponsive polymer/IL composites, in which the temperature response originates from a polymer such as PNIPAM, should also be distinguished from TRILs [88]. In general, TRILs enable the reversible restructuring of the carrier’s microstructure by dynamically regulating hydrogen bonds, ionic interactions, and hydrophobic association through temperature control, thereby triggering “switch-like” controlled drug release. At the same time, ILs can enhance the solubility of poorly soluble small-molecule drugs, peptides, and nucleic acid-based drugs, achieving an integrated approach of solubilization and temperature-controlled release. In recent years, drug delivery by TRILs has garnered widespread attention and is applied in abundant areas, such as localized cancer drug delivery, transdermal delivery, injectable in situ gels, and stable carriers for protein-based drugs [89]. Liu et al. designed and constructed a thermodynamically stable IL-based microemulsion (IL-M), which integrates the high drug solubility of ILs and high skin permeability of microemulsions, thus improving the local delivery of copper peptides by approximately three-fold while retaining their biological function [80]. Experiments in mice validated the effectiveness of our proposed IL-M system. Nevertheless, unresolved challenges for TRILs applying in drug delivery, such as potential cytotoxicity, leakage of small-molecule ILs, and insufficient controllability of release kinetics, impede further clinical translation. Future research should focus on the design of biocompatible and degradable TRILs, fabrication of multi-stimuli-responsive composite carriers, and systematic in vivo pharmacological evaluation to advance the development of precision therapeutics.

### 4.4. Catalysis

Homogeneous catalysis features high catalytic activity and exceptional selectivity, yet suffers from inherent drawbacks including difficult catalyst-product separation, tedious recovery procedures, and precious metal leaching [90]. In contrast, heterogeneous catalysts facilitate simple separation but are commonly limited by insufficient exposure of active sites and severe mass transfer resistance. As emerging temperature-responsive smart media, TRILs exhibit reversible LCST/UCST phase transitions via rational cation-anion structural modulation and enable a temperature-switchable phase-separation catalytic system, integrating the merits of homogeneous catalysis (high-temperature homogeneous reaction) and heterogeneous catalysis (low-temperature phase separation and catalyst recovery) [91]. The mechanism of TRIL-based catalytic systems originates from temperature-driven reorganization of microscopic phase behavior. For LCST-type small-molecule TRIL-based thermomorphic biphasic systems, mutual miscibility between the ionic liquid and reaction medium occurs once the temperature exceeds the critical point, generating a homogeneous reaction environment. Catalytic active sites are well dispersed, enabling sufficient contact between substrates and catalysts and thereby accelerating reaction kinetics. After reaction completion, cooling below the phase transition temperature induces spontaneous liquid–liquid phase separation, wherein catalysts are concentrated within the ionic liquid phase [80]. Catalyst separation from organic products can be readily realized via simple settling, phase stratification and decantation. By contrast, UCST-type systems display inverse behavior, with precipitation at low temperatures and dissolution at elevated temperatures. Most importantly, the phase transition temperature, the core parameter, can be precisely tuned to match the operating window of target reactions by adjusting the alkyl chain length of cations, anion species and polar side-chain substituents.

In the fields of organo-catalysis and CO_2_ valorization, TRIL catalysts exhibit remarkable application potential, which can not only serve simultaneously as reaction media and organo-catalysts, but also synergistically activate epoxides, thereby catalyzing the cycloaddition of CO_2_ and epoxides to produce cyclic carbonates. Gao et al. reported the first application of the pyridine-based IL [MPY][DMP] in a DMC/MT azeotropic separation system [81]. Through the COSMO-RS high-throughput screening, vapor–liquid equilibrium experiments, molecular dynamics simulations, and the design of a heat-pump-assisted extractive distillation process, this study systematically elucidated the mechanism of selective hydrogen bonding between the anion [DMP]^−^ and methanol, and reduced total annual cost (TAC) and total energy consumption (TEC) by 8.27% and 48.68%, respectively, compared to traditional extractive distillation processes. In transition metal-coupled catalytic systems, TRILs act as smart supports for noble metal nanoparticles, where temperature modulates the hydrophilicity/hydrophobicity and aggregation state of the supports, further regulating the interfacial microenvironment of nanocatalysts. This strategy achieves temperature-dependent selectivity switching in tandem reactions, such as Suzuki coupling and asymmetric transfer hydrogenation. Selective activation of specific reaction pathways via temperature manipulation permits directional product regulation within one-pot systems, simplifying multi-step synthetic routes [82].

### 4.5. Electrochemical Energy Storage

Conventional aqueous and organic electrolytes in electrochemical energy storage devices suffer from inherent drawbacks of flammability, volatility, narrow electrochemical windows and poor high-temperature stability [92]. TRILs are capable of reversible LCST/UCST phase transitions via rational cation-anion engineering, which can convert temperature signals into reversible changes in ion transport, microscopic phase structure, and interfacial impedance, providing a novel dielectric solution for the development of temperature-controlled, self-protecting smart energy storage devices. In general, the regulation mechanism of TRIL-based energy storage systems originates from temperature-induced evolution of intermolecular interactions and microphase structures [85]. For LCST-type TRIL electrolyte systems, favorable compatibility between ILs and polymer matrices can construct continuous ion transport pathways below the phase transition temperature and retain high ionic conductivity, enabling normal energy storage operation of devices. When the temperature rises above the critical phase transition temperature, microphase separation occurs because of the weakening of hydrogen bonding and the intensification of hydrophobic interactions. Furthermore, polymer aggregates block ion migration channels and cause a sharp increase in charge transfer resistance, which effectively suppresses charging and discharging reactions. Oppositely, UCST-type systems exhibit a different behavior. A homogeneous state with high ionic conductivity is maintained at elevated temperatures, whereas cooling induces phase separation to interrupt ionic conduction [93]. By tuning the alkyl chain length of cations, anion species and polymer proportions, the phase transition temperature can be precisely adjusted to the safety warning range, achieving reversible overheating protection for energy storage devices. Hence, IL-based systems for electrochemical energy storage feature negligible volatility, excellent low-temperature resistance and anti-desiccation performance, rendering them more suitable for long-duration energy storage under wide temperature windows.

Thermoresponsive ionogel electrolytes have been extensively explored for safety-oriented supercapacitors and flexible energy storage devices. Many researchers fabricated the ionogels by confining small-molecule TRILs within three-dimensional polymer networks, which integrate the high electrochemical stability of ILs with the leakage-free and shape-tunable merits of gel materials. At ambient temperature, continuous conductive networks form inside the ionogel, and the assembled supercapacitors deliver stable specific capacitance [94]. Once the temperature exceeds the preset threshold, thermally induced phase separation blocks ionic transport pathways, leading to substantial capacitance decay or even quasi-open-circuit states, thereby realizing reversible self-protection against overheating. Benefiting from the outstanding flexibility of ionogels, separator-free monolithic flexible supercapacitors can be constructed to satisfy wearable applications while incorporating thermal safety functionality. Furthermore, the stepwise variation in ionic conductivity across the phase transition facilitates the development of thermally switchable energy storage devices, enabling programmable temporal regulation of energy storage and release. Peng et al. provide an effective strategy combining thermosresponsive hydrogel electrolytes with electrochromic pseudocapacitive electrodes, which is proposed to address thermal runaway risks, enabling smart supercapacitors (SSCs) integrated with monitoring, self-protection and early-warning functions [93]. Specifically, the synthesized PNAB gel electrolyte exhibits a loose porous network populated by numerous macropores with average diameters larger than 5 μm (open state) at 20 °C, and therefore retaining abundant aqueous electrolyte, facilitating ion migration, and endowing the SSC with high transmittance. In contrast, upon heating to 65 °C, all initial macropores are tightly closed, revealing that the PNAB gel network begins to shrink and expel adsorbed aqueous electrolyte. Further elevating the temperature to 95 °C completely eliminates these pores and generates a dense polymer layer. Therefore, the ionic conductivity of the electrolyte decreases drastically upon heating, enabling self-protection of smart supercapacitors once thermal runaway occurs.

Despite the promising prospects of TRIL-based smart energy storage systems, their practical deployment is still restricted by multiple bottlenecks. Most TRILs suffer from high viscosity and relatively low ionic conductivity under ambient conditions, whereas small-molecule ionogels inevitably encounter gradual IL leakage during long-term cycling. Moreover, the broad phase-transition temperature range fails to achieve precise and sharp thermo-switching behavior, and the interfacial compatibility and electrochemical stability between ILs, electrodes and lithium salts under high-temperature cycling remain insufficiently verified. Future research should focus on the rational design of low-viscosity TILs with wide electrochemical windows and accurately tunable phase-transition temperatures, the fabrication of all-polymerized ionic liquid crosslinked gels, and the construction of multi-stimuli responsive energy storage systems. Clarifying the structure–performance relationship between phase transition behavior and electrochemical stability will further promote the practical application of high-safety and adaptive energy storage devices.

## 5. Conclusions

This review systematically summarizes the structural features, thermally induced phase transition mechanisms, and multidisciplinary applications of temperature-responsive ionic liquids (TRILs). The unique thermo-switchable performance of TRILs originates from temperature-regulated dynamic variations in intermolecular interactions, which can be precisely tailored via rational cation-anion structural engineering. Benefiting from tunable LCST/UCST liquid–liquid transition and diverse liquid–solid transition behaviors, including precipitation, crystallization, liquid crystal formation, and plastic crystal formation, TRILs exhibit excellent environmental adaptability and versatile application potential in water treatment, gas separation, controllable drug delivery, switchable catalysis, and electrochemical energy storage, effectively addressing the inherent limitations of conventional static functional materials through intelligent phase-transition-induced functional switching. Despite these substantial advances, the large-scale practical implementation of TRILs is still constrained by inherent drawbacks such as high viscosity, low ionic conductivity, inevitable small-molecule leakage, wide phase transition ranges and unsatisfactory long-term interfacial stability, while their biocompatibility, environmental safety and industrial economic feasibility also require further systematic verification and optimization. Future research should focus on the rational design of low-viscosity, low-cost and biocompatible TRILs, the construction of all-poly (ionic liquid) composite systems and multi-stimuli-responsive platforms, and the in-depth clarification of structure–activity relationships between phase transition behavior and functional performance. Continuous optimization of material structures and composite systems will accelerate the industrial translation of TRILs and offer innovative insights for the development of high-efficiency, safe and intelligent chemical and energy technologies.

## Figures and Tables

**Table 1:** Representative TRIL-based applications.

Application Area	TRILs System	Phase Transition Type	Key Performance	References
Water treatment	[P_4444_][DMBS]	LCST	Osmotically assisted RO for high-salinity wastewater concentration	Lee et al. [75]
Water treatment	[choline][NTf_2_]/[OH]	LCST	Extraction at 25 °C and phase separation at 80 °C	Durling et al. [13]
Water treatment	PNIPAM	LCST	Temperature-switchable Cu^2+^ adsorption at 40 °C, and regeneration at 20 °C	Sun et al. [76]
Gas separations	Amino-functionalized ILs	Dissolution–precipitation	Selective separation of aromatics and alkanes	Jiang et al. [77]
Gas separations	Amino-functionalized IL + [Bmim][BF_4_]	Dissolution–precipitation	Up to 90% CO_2_-rich phase; reduced regeneration energy	Asensio-Delgado et al. [78]
Gas separations	Carboxylic acid IL (CAILs) in PC	Dissolution–precipitation	CO_2_ cycloaddition with epoxides	Meng et al. [68]
Drug delivery	[Ch][Ket]	LCST	Transdermalrateis 1.4–2.2 timesthatofketoprofengel.	Tang et al. [79]
Drug delivery	IL-based microemulsion (IL-M)	LCST	3-fold improved local delivery of copper peptides	Liu et al. [80]
Drug delivery	Pyridine-based IL [MPY][DMP]	LCST	DMC/MT azeotropic separation; reduced TAC and TEC	Guo et al. [81]
Drug delivery	LATTE IL ablative agent	LCST	mage-guided local injection; uniform drug distribution; tumor ablation	Rahman et al. [82]
Catalysis	Ru-Pd/ILs	LCST	Tunable catalytic performance in asymmetric aldol addition reaction	Kuepfert et al. [83]
Electrochemical energy storage	PNAB hydrogel ILs	LCST	Smart supercapacitor with monitoring, self-protection, and early-warning functions	Wang et al. [84]
Catalysis	Zinc-Cyclen ILs	LCST	Turnover rates of 2.02 × 10^5^ M^−1^ s^−1^ at 25 °C and 1.69 × 10^6^ M^−1^ s^−1^ at 75 °C,	Malviya et al. [85]
Electrochemical energy storage	EMIBF4	LCST	Work stably at 3 V, 45 °C for 1500 h	Hirukawa et al. [86]

## Data Availability

No new data were created or analyzed in this study. Data sharing is not applicable to this article.
